# Characterization of aged male BALB/c_cenp_ mice as a model of dementia

**DOI:** 10.1186/s42826-020-00038-0

**Published:** 2020-03-05

**Authors:** Nashelly Esquivel, Yenela García, Bestraida Lores, Marivy Gutiérrez, Claudio Rodríguez

**Affiliations:** 1 Laboratorio de Antianémicos y Nutracéuticos, Centro Nacional de Biopreparados (BioCen). Beltrán main road km 1 1/2, Mayabeque, Cuba; 2 Department of Patology, Experimental Toxicology Center (CETEX), Centro Nacional para la Producción de Animales de Laboratorio (CENPALAB), 3rd street, N° 40759 between 6th street and Tirabeque main road, Havana, Cuba

**Keywords:** Dementia, Alzheimer’s type dementia, Animal models, Aging

## Abstract

Dementia is defined as cognitive impairment in more than one cognitive area and leads to an abnormal degree of impairment in the ability to remember past events. Among mice models of dementia the most used strains are SAMP8 and C57BL/6. There is no reference to characterizing a model of dementia in naturally aged mice of the BALB/c strain, or to the minimum age at which these animals can be used. The aim of this study was the characterization of aged male BALB/c_cenp_ mice as a model of dementia from the evaluation of behavioural, pathological and biochemical markers. One hundred and twenty mice were used and 10 of these were analysed from 8 to 9 months of age, and every 4 months, in a comparative way to young control animals from 4 to 5 months. At the age of 12–13 months there was cognitive impairment in the animals from the Y-maze and object recognition tests and this impairment was maintained at 16–17 months of age. An increase in oxidative damage to proteins in the brains of aged animals was also found in relation to young animals; as well as a decrease in the concentration of triglycerides. At the age of 16–17 months, a significant decrease in the size of the thymus and brain was obtained. We consider that it’s a very useful option to use animals 12–13 months of age where there are symptoms of cognitive deficiency, histopathological and biochemical elements characteristic of dementia.

## Introduction

Dementia is defined as cognitive impairment characterized by loss of intellectual ability of sufficient severity to interfere either with occupational functioning and usual social activities [1]. Cognitive areas involved in dementia includes aphasia (language deterioration), apraxia (motor difficulties), agnosia (failure to recognize objects despite intact sensory capacity), or a disturbance in executive functioning [2]. Around 80% cases of dementia worldwide are represented by Alzheimer’s disease (AD) and they are characterized by different stages of cognitive and functional impairments [3].

AD is characterized histopathologically by extracellular amyloid-beta plaques and intraneural fibrillar tangles due to tau protein hyperphosphorylation, oxidative stress and synaptic and neuronal losses [4]. The gradual development of AD is due to the action of different pathophysiological events, among which the activation of innate immunity in the central nervous system, mainly by microglial cells, that is a key element in the neurodegenerative process [5].

Development of animal models of dementia is challenging as there is no single animal model that can explain all the cognitive, behavioural, biochemical and histopathological abnormalities of different types of this pathology [6]. An ideal animal model should mimic the human disease and reproduce complexities of human behaviour in rodents. Several animal models of AD and cognitive impairment have been developed, which can be broadly classified according to diverse pathophysiological bases [7]. To develop animal models of dementia generally use transgenic animals or the bilateral stereotaxic administration into the ventricles of the brain of different chemical substances. However, the major disadvantage of these models is that the acute phase of the disease doesn’t reproduce the pathology as it evolves in humans, where the development of disease occurs over months and years. Added to these disadvantages to prove some hypotheses, is the high cost of transgenic animals that increase the price of neurobiological research [8].

In front of this situation some studies report the use of economic models, non-invasive and that reproduce part of the pathogenesis of the disease. Aged animals are used routinely in drug development due to age related cognitive decline and behavioural alterations which mimic not only the neurochemical and morphological alterations but also the cholinergic hypofunction that is similar to pathophysiology of AD [9–11]. Another advantage of this model is that they develop without any central neurochemical manipulations and occurs a slow progression of the disease associated to the natural aging process [12]. Rodents have a leading role in animal models of dementia and among the most used strains by researchers studying cognitive disorders are C57BL/6 [13] and SAMP8 [14]. In the case of the BALB/c strain there are few studies where it was used aged mice as a model of dementia [15].

There is no reference to the characterization of a dementia model in aged mice of the BALB/c strain, even though it’s widely used in behavioural studies related to stress, fear and cognitive function [16–18]. On the other hand, among the models of aged mice described for dementia there is variability about the minimum age at which behavioural, pathological or biochemical symptoms of the disease take place [13–15]. In all of these models there is great variety about the minimal age at which behavioural, pathological or biochemical symptoms of the disease appear. Therefore it’s important to characterize a model where these aspects of dementia are explained and centralized. The aim of this study was the characterization of aged male BALB/c_cenp_ mice as a model of dementia from the evaluation of behavioural, pathological and biochemical markers.

## Materials and methods

### Animals and housing conditions

Young BALB/c_cenp_ male mice (20–24 g, *n* = 120) were purchased from Centro Nacional para la Producción de Animales de Laboratorio (CENPALAB, La Habana, Cuba). All experimental mice were given space with a controlled consistent temperature (21 ± 3 °C) and lighting environment (12 h/12 h light/dark cycle). Mice were fed with the EAO1004_cenp_ diet for rodents ad libitum and drinking water. The animals were adapted for a week to the experimental conditions prior to the beginning of the experiments and then mice were individual identified by ears perforation nomenclature. Animals were placed in polypropylene cages with wood chip bedding (Sournid, Spain), at the rate of 10 animals per cage. All experimental protocols related to the use of animals were approved by the Institutional Animal Care and Use Committee at Centro Nacional de Biopreparados.

### Experimental design

When the animals were 4–5 months of age a sample of 10 animals was randomly selected and they were considered the control group. From this first experimental time, groups of 10 animals were randomly selected every four months until animal showed cognitive impairment. Body weight was measured in all the experimental times, and cognitive function was evaluated by the application of different behavioral tests. In addition percent survival and the percentage of animals that showed an adequate state of health were determined from the total of animals that started the experiments in each experimental time.

### Behavioral tasks

The animals were transferred to the place where the behavioral tests were carried out 24 h before the beginning of the assays. At the end of each task, the surfaces of the mazes were disinfected with 70% ethanol solution.

Y-maze task (spontaneous alternation): it allows evaluating the spatial memory of short-term work [19] and in this study it was used the methodology described by García and Esquivel [20]. In the spontaneous alternation task, it was considered that the mice made alternations when they sequentially visited the three arms, without repeating any. We also counted the number of repeated entries to the same arm (perseveration) and incorrect entries to the arms for each animal. Finally, spontaneous alternation was calculated by the following expression:

$$ \mathrm{Spontaneous}\ \mathrm{alternation}\ \left(\%\right)=\frac{Alternations}{Total\ of\ \mathrm{p} ossible\ alternations} \times 100 $$

Total of possibles alternations = Total of entries - 2.

Object recognition task: it’s based on the natural instinct of rodents to explore novel objects and evaluate non-spatial memory [21]. The task was performed under the conditions described by García and Esquivel [20].

The percentange of time that mice spent recognizing the novel object (NO) or familiar object (FO), relative to the total object recognition time or Preference Index (PI), was calculated by the following expression:

$$ \mathrm{IP}\ \mathrm{NO}\ \left(\%\right)=\frac{Time\  Exp. NO}{Time\  Exp. FO+\mathrm{Time}\ \mathrm{Exp}.\mathrm{NO}}\times 100 $$

Time Exp. FO: Time of exploration of FO

Time Exp. NO: Time of exploration of NO

### Hematological and biochemical determinations

Ten animals from 8 to 9 months and 12–13 months of age and 10 control animals from 4 to 5 months of age were used. The animals were anesthetized in a ketamine and xylazine (Sigma-Aldrich, Merck, Darmstadt, Germany) mix and approximately 100 μL of blood was extracted by the retro-orbital plexus, which was added the ethylenediaminetetraacetic acid (EDTA) anticoagulant solution (Sigma-Aldrich, Merck, Darmstadt, Germany). This sample was analyzed in the optical microscope (Olympus, Tokyo, Japan), with the objective of 100 × immersions, and 100 cells were observed to calculate the percentages corresponding to the nuclear polymorphs (NPM) and the lymphocytes. The rest of the blood was then collected without EDTA (Sigma-Aldrich, Merck, Darmstadt, Germany) to obtain the serum. In the serum sample collected, the concentration of triglycerides was measured with the Monotriglitest reagent kit (HELFA® Diagnostics, La Habana, Cuba). It was also obtained brain homogenates in Tris buffer at pH 7.6 containing 0.32 mM sucrose (Sigma-Aldrich, Merck, Darmstadt, Germany), 10 mM Tris (Sigma-Aldrich, Merck, Darmstadt, Germany) and 1 mM EDTA (Sigma-Aldrich, Merck, Darmstadt, Germany) and differential centrifugation was performed to obtain the post-mitochondrial fraction, according to the method described by Uwe and Von Hagen [22]. The supernatant was dispensed in aliquots of 1 mL, which were stored at − 80 °C. In these homogenates the oxidative damage to lipids and proteins was measured.

The oxidative damage to the lipids was measured from the thiobarbituric acid reactive substances (TBARS) method according to Okawa et al. [23]. The TBARS were determined from the molar extinction coefficient of the malondialdehyde (MDA) (ξ = 155 mM - 1 cm - 1 mL). The concentration of proteins in the tissue homogenate was determined by the modified Lowry method for biological tissues [24]. In addition, to quantify the oxidative damage to proteins, it was used the method described by Resnick and Parker [25]. The amount of proteins with carbonyl groups was determined from the maximum absorption measured at 360 nm; and the molar extinction coefficient of hydrazine (ξ = 22.000 mM - 1 cm - 1 mL). On the other hand, the concentration of proteins in the samples was calculated from different concentrations of bovine serum albumin (0.2 to 2.0 mg/mL) that were read at 280 nm. Finally, the result was expressed as the relationship between the molar concentration of the carbonyl groups and the concentration of proteins present in the tissue homogenate.

### Histological studies

Histological analyzes were performed in 5 young animals (4–5 months of age) and 5 aged animals (16–17 months of age). The animals were sacrificed by cervical dislocation and the brain, thymus, spleen, liver, kidneys and lungs were colected. Organs were rinsed with cold 0.9% NaCl solution (Sigma-Aldrich, Merck, Darmstadt, Germany) (2–8 °C) to eliminate rest of blood, dried with filter paper and were weighted using an analytic balance (Sartorius, Göttingen, Germany). The relative weight of the organ was calculated in relation to the body weight of the animal before sacrifice (also was analized weight organs in the esperimental times of 8–9 and 12–13 months of age). All mice organs, except the thymus, were fixed in phosphate buffered 10% formalin solution (Sigma-Aldrich, Merck, Darmstadt, Germany). After inclusion in paraffin, the organs were cut and stained with hematoxylin/eosin (Sigma-Aldrich, Merck, Darmstadt, Germany) according to conventional techniques. The histological evaluation of organs and tissues was carried out with an optical microscope (Meiji Techno, Tokyo, Japan).

### Statistical analysis

One-way analysis of variance (ANOVA) was used to identify significant differences between all experimental groups (GraphPad Prism 5 for Windows, Release 5.03, Standard Version, San Diego, CA, USA). These levels were verified by Tukey’s post-hoc analysis. All values were expressed as the means ± standard deviation (SD) and a *P* value < 0.05 was considered significant.

## Results

### Percent survival and body weight

Percent survival of male BALB/c_cenp_ mice at the age of 16–17 months was 93.33%. However, 51 animals were sacrificed (42.50% of the total) due to a deteriorated health state when the mice reached the age of 8–9 months. The sum of the natural deaths and the animals that were sacrificed represented a real survival of 50.83%.

**Fig. 1:**
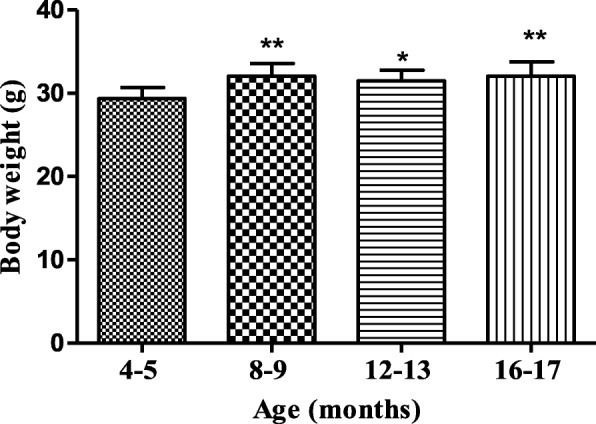
Body weight of male BALB/c_cenp_ mice up to 16–17 months of age. Data shown are the means±SD. * indicates *P* < 0.05 compared to young control group (4–5 months of age)

The body weight of male BALB/c_cenp_ mice showed statistically significant differences from the age of 8–9 months, whose weight was significantly greater than that of the control group from 4 to 5 months (Fig. 1). Among the aged mice groups there were no statistically significant differences.

### Behavioral tasks

**Fig. 2:**
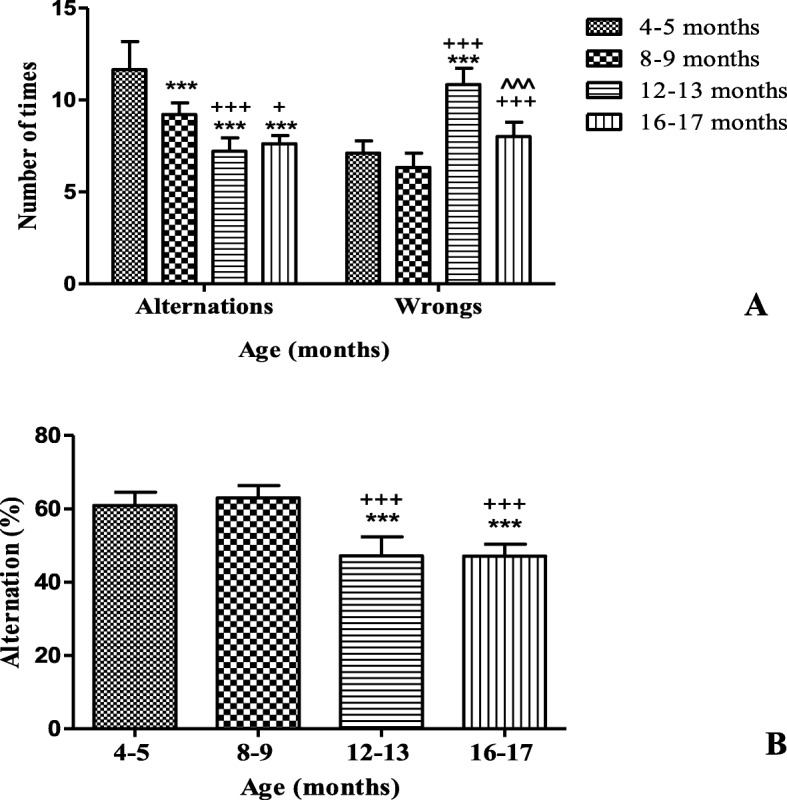
Y maze task of male BALB/c_cenp_ mice up to16–17 months of age. **a** Number of alternations and wrongs when that the animal visit each arm. **b** Percentage of alternation. Data shown are the means±SD. * indicates *P* < 0.05 compared to young control group (4–5 months of age). + indicates *P* < 0.05 compared to group of 8–9 months of age. ^ indicates *P* < 0.05 compared to group of 12–13 months of age

As a result of the tasks that were applied to evaluate the cognitive function, it was obtained in the Y maze that there were significant differences in relation to the number of alternations obtained in the different experimental times (Fig. 2a). The differences were found between the groups of animals 8–9, 12–13 and 16–17 months of age, in relation to young animals group. In addition, significant differences were found in the number of errors (incorrect entries to the arms of the Y maze) where the group of 12–13 months of age differed statistically from the rest of the groups. The percentage of alternation was significantly lower in the animals from 12 to 13 and 16–17 months of age than in the animals from 8 to 9 and 4–5 months of age (Fig. 2b).

**Fig. 3:**
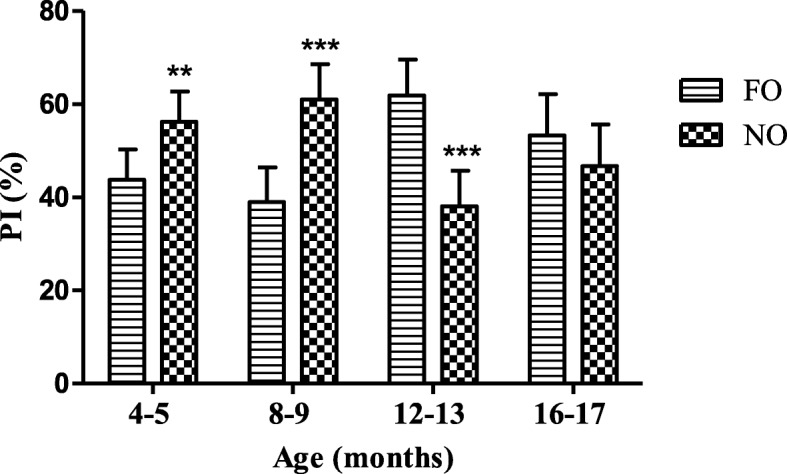
PI (preference index) for FO (familiar object) and NO (novel object) in the object recognition task of male BALB/c_cenp_ mice up to 16–17 months of age. Data shown are the means±SD. * indicates *P* < 0.05 compared to FO at each group

The other behavioral task used to evaluate if the naturally aged BALB/c_cenp_ mice had cognitive impairment was the object recognition. In this task there were obtained significant differences between the PI by FO and NO in each of the ages of the animals evaluated, except for mice of 16–17 months of age (Fig. 3). In the mice of 4–5 and 8–9 months of age PI by NO was significantly higher than by FO. However, in group of 12–13 months of age PI was significantly higher for FO than for NO.

### Hematological and biochemical determinations

**Fig. 4:**
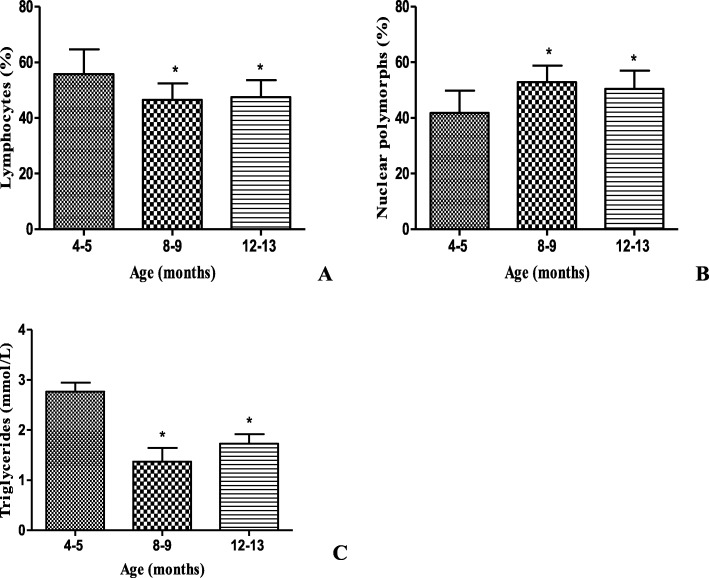
Hematological parameters and triglyceride concentration in male BALB/c_cenp_ mice up to 12–13 months of age. **a** Lymphocytes. **b** Nuclear polymorphs. **c** Triglycerides. Data (*n* = 10) shown are the means±SD. * indicates *P* < 0.05 compared to young control group (4–5 months of age)

One of the hematological parameters analyzed was the population of lymphocytes that in the group of 12–13 month of age decreased significantly in relation to the young animals (Fig. 4a). On the other hand, the population of nuclear polymorphs (Fig. 4b) in animals 12–13 months of age was significantly higher than in animals from 4 to 5 months of age. The concentration of triglycerides in the serum showed a significant decrease in the group of 12–13 month of age in comparison with the animals of 4–5 months of age (Fig. 4c).

**Fig. 5:**
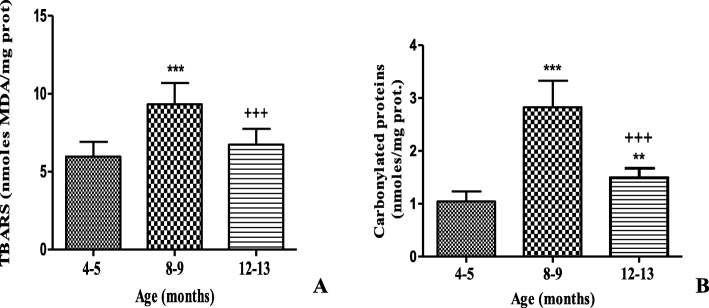
Oxidative damage in brain homogenate of male BALB/c_cenp_ mice up 12–13 months of age. **a** TBARS (thiobarbituric acid reactive substances) as an indicator of damage to lipids. **b** Carbonylated proteins as an indicator of damage to proteins. Data shown are the means±SD. * indicates *P* < 0.05 compared to young control group (4–5 months of age). + indicates *P* < 0.05 compared to group of 8–9 months of age

Also in these male BALB/c_cenp_ mice at the age of 12–13 months, the oxidative damage to the lipids in the brain homogenate had an increase nevertheless those there wasn’t a significant difference in relation to the animals of 4–5 months of age (Fig. 5a). On the other hand, oxidative damage to proteins showed a significant increase in animals of 12–13 compared to animals of 4–5 months of age (Fig. 5b).

**Fig. 6:**
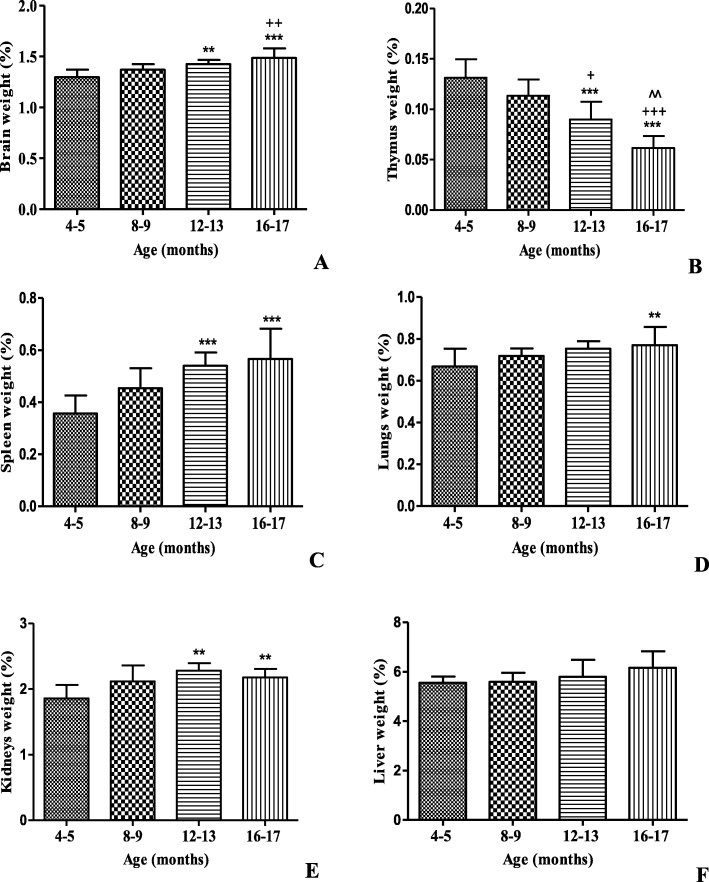
Weight of the organs relative to the body weight of male BALB/c_cenp_ mice up to 16–17 months of age. **a** Brain weight. **b** Thymus weight. **c** Spleen weight. **d** Lungs weight. **e** Kidneys weight. **f** Liver weight. Data shown are the means±SD. * indicates *P* < 0.05 compared to young control group (4–5 months of age). + indicates *P* < 0.05 compared to group of 8–9 months of age. ^ indicates *P* < 0.05 compared to group of 12–13 months of age

It was found that weight of brain in mice of 16–17 months of age was significantly lower than in the control mice (Fig. 6a). In the case of the thymus, the weight was significantly lower in the animals at the age of 16–17 months than in the controls group (4–5 months of age) (Fig. 6b). On the other hand thymus weight was 22% lower than the weight in the control animals. As a result of the comparison between the groups for weight of spleen, lungs and kidneys, was obtained a significant increase in the animals of 16–17 months of age in relation to control group (Fig. 6c-g). Liver weight didn’t show significant differences between the experimental groups (Fig. 6f).

### Histological studies

**Fig. 7:**
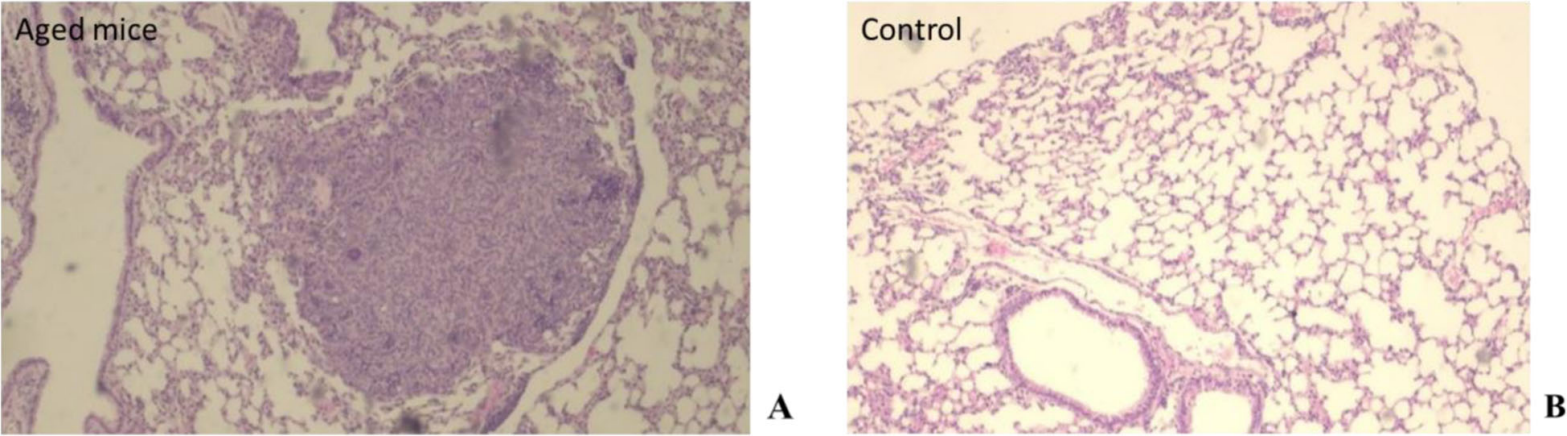
Damages presented in lung of BALB/c_cenp_ male mice of 16–17 months of age **a** Focal lesion with tumor appearance present in the lungs of mice of 16–17 months of age. **b** Lungs of control group (4–5 months of age). HE 100×

Among the histological studies, the morphology of the lung was evaluated and inflammatory infiltrative alterations and aberrations of cell growth were observed. The most visible finding was the thickening of the alveolar septa, characterized by a minimal infiltration of lymphocytes and few macrophages; it was presented in 40% of the animals of the group aged of 16–17 months of age and in 20% of those of the young group. In addition, in 40% of the aged animals, anaplasias with a focal character were observed in the lung, well delimited, rounded, characterized by cellular polymorphism, presence of mitotic details in the nuclei of some cells of the periphery and poor vascularization (Fig. 7).

**Fig. 8:**
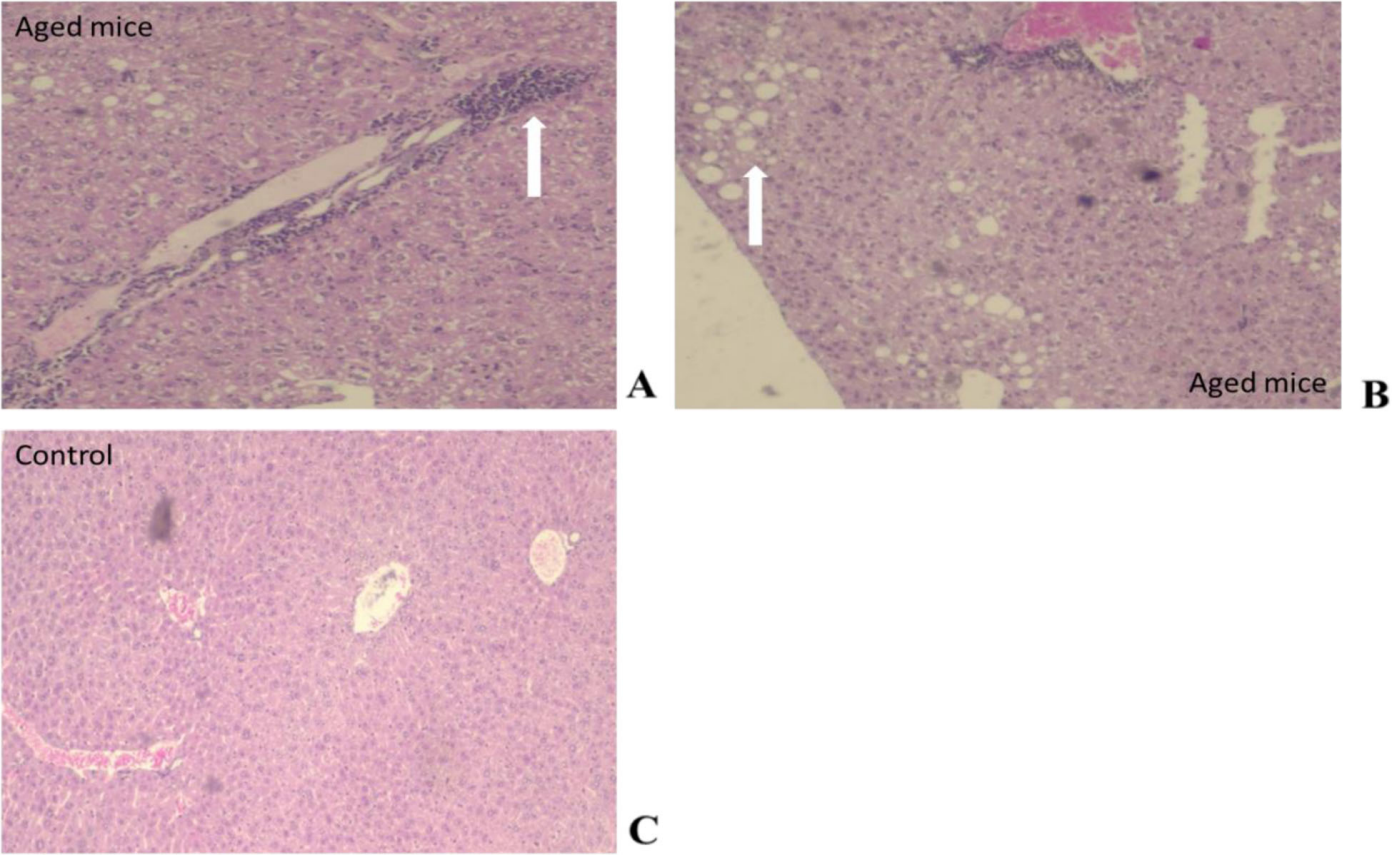
Damages presented in liver of BALB/c_cenp_ male mice of 16–17 months of age. **a** Perivascular lymphocytic infiltration. HE 40×. **b** Macro focal and micro vesicular lipidosis. **c** Liver of control group (4–5 months of age). HE 200×

Few liver glycogen was observed in the liver of 20% of young animals. However, the most relevant alterations occurred in 60% of the aged animals and were the discrete perivascular lymphocytic infiltration (Fig. 8a) and focal lipidosis (Fig. 8b). Steatosis was present in 60% of the animals in the group of 16–17 months of aged.

**Fig. 9:**
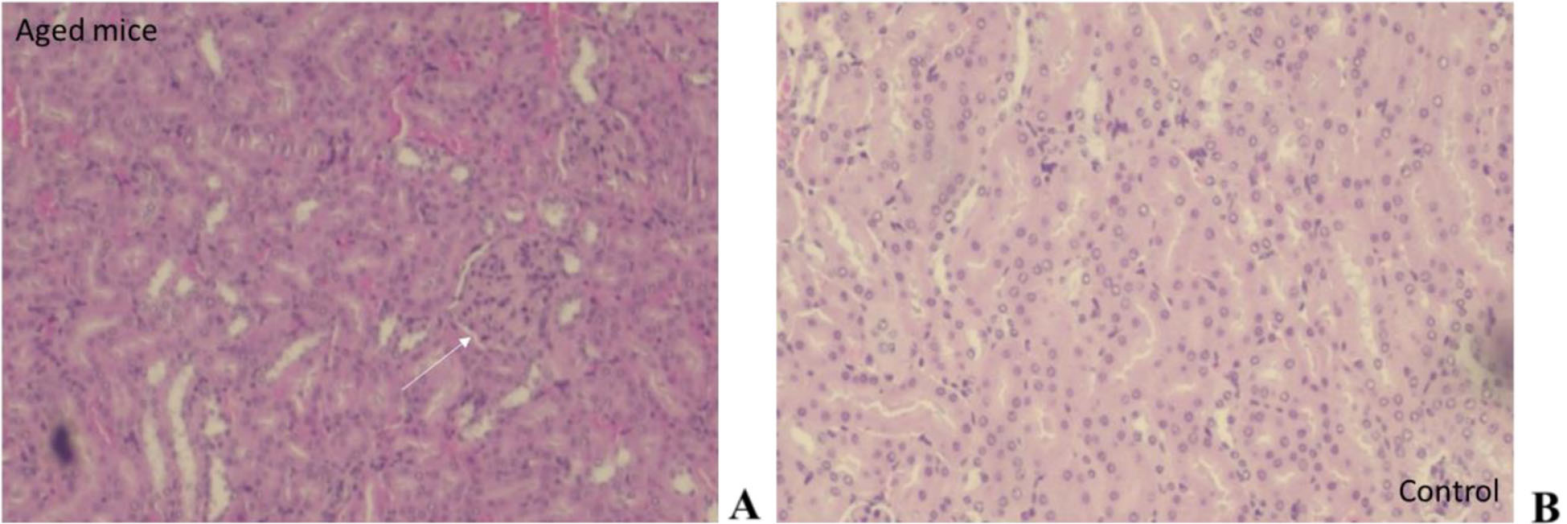
Damages presented in kidney of BALB/c_cenp_ male mice of 16–17 months of age. **a** Degeneration of the glomeruli. HE 200X. **b** Kidney of control group (4–5 months of age). HE 200×

**Fig. 10:**
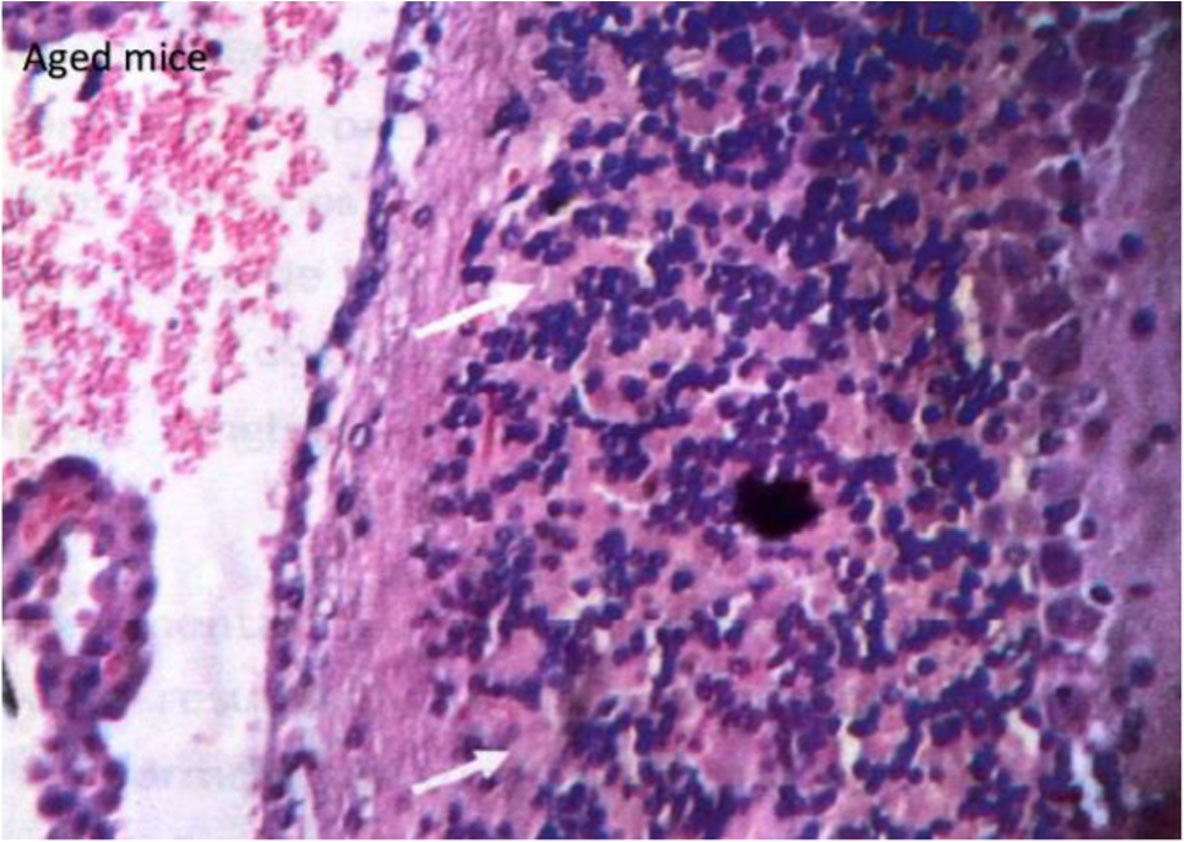
Degeneration of cerebellum’s granular layer of BALB/c_cenp_ male mice of 16–17 months of age. HE 200×

The findings observed in the kidney and in the brain were only in the group of animals of 16–17 months of age. In the case of kidney, the most important damage presented was a glomerular degeneration, characterized by a decrease in its capillaries (Fig. 9a). In the case of brain, in 40% of animals aged 16–17 months, an area with degeneration of the granular layer of the cerebellum was observed (Fig. 10). On the other hand, the morphological characteristics of the hippocampus were normal for both young control animals and those aged 16–17 months of age.

## Discussion

Percent survival obtained at the end of the experimental procedure and the number of mice sacrificed due to their deteriorated state of health, were due to the frequent fights produced between the animals of the same cage. Although there isn’t reference about the survival percentage of BALB/c aged mice. However the occurrence of frequent brawls in the cages is due to the fact that the male BALB/c are the most aggressive strain of mice and those with greater difficulty to adapt to group housing [26].

When the mice were 8–9 months of age the aggressiveness reached the greatest dimensions during the experimental period. In consequence of this situation mice were housed individually. In accordance with several studies, an increase in mice body weight until 8–9 months of age was observed and after this experimental time body weight then remains constant (Fig. 1) [27–29].

The results obtained in the Y-maze test showed the superiority for the control group in the number of alternations (Fig. 2a) in relation to animals of 8–9, 12–13 and 16–17 months of age. Significant decrease in the percentage of alternation (Fig. 2b) was found in the animals from 12 to 13 and from 16 to 17 months of age. These results suggest that aged animals, from 12 to 13 months of age, show a decrease in the ability to orient themselves in the space and they don’t show the natural instinct to explore new places that is reported for young male mice of 9 weeks of age without being subjected to any drug or treatment [19, 30]. Therefore, based on the mechanism of memory that is evaluated in the Y-maze test, it can be affirmed that male BALB/c_cenp_ mice since aged of 12–13 months show cognitive impairment that appears in dementia.

The results obtained in the object recognition test (Fig. 3) allow us to affirm that when the animals are 12–13 and 16–17 months of age, the motivation to explore new objects isn’t activated correctly, which indicates the appearance of cognitive impairment [31]. The fact that in this work the cognitive impairment of BALB/c_cenp_ aged mice is demonstrated with the application of two behavioural task, Y maze and object recognition test (Figs. 2 and 3) that are related to hippocampal functionality [32], demonstrates the usefulness of this animal model for the study of dementia and AD.

Contrary with the reported by literature about the changes in the population of NPM (Fig. 4b) [33], it was found that in the mice from 12 to 13 months of age there was a significant increase compared to the young control animals. However according to Shaw et al., [34] the balance between the generation of myeloid lineages (monocytes and granular leukocytes, such as NPM, neutrophils, basophils and eosinophils) and lymphoid (T, B and NK lymphocytes), characteristic of young individuals, is lost with aging (Fig. 4a, b), showing a tendency towards myeloid progenitors to the detriment of lymphoid. In the case of the lymphoid fraction of the animals from 12 to 13 months of age (Fig. 4a), it supposes that these decreases are related to the thymus involution that occurs during aging, and in particular, the progressive loss of the thymic epithelial space, where the thymopoiesis takes place [34].

In the case of the thymus weight, this behaviour has been corroborated in naturally aged mice, specifically BALB/c, where the involution of the organ started from 12 weeks of age (3 months). These authors also found that at 35 weeks (8–9 months) the weight decreased by 45% in relation to the weight of the organ when the animal was 6 weeks old [35].

In agreement with studies in CBA/C a mice and in rats, our data reveal a strong increase in spleen weight (Fig. 6c) until the age of 16–17 months [36]. Some studies have shown age-related white pulp atrophy in the spleen of Sprague–Dawley rats which correlate with a decrease of greater than 80% in lymphocytes number with aging [37]. The results obtained in this work with relation to weight of thymus and spleen (Fig. 6b, c) may explain the decrease in the lymphocyte population obtained in aged mice (Fig. 4a) because in these organs take place lymphocyte production.

The result obtained in this work about serum triglycerides concentration (Fig. 4c) is agree with those obtained by Araki et al. [38]. According to these authors, in aged animals the mobilization of lipids deteriorates with age due to the decrease in the gene expression of Apos. This leads in the long term to an excessive accumulation of lipids in tissues such as the liver, resulting in a higher incidence of age-related diseases and in particular dementia. Morever, the focal lipidosis in liver obtained in BALB/c_cenp_ mice at 16–17 month of age (Fig. 8b) suggest the presence of an increase of circulating lipids that is very possible to include triglycerides. In the liver, aging causes an increase in lipid accumulation (micro and macro vesicular lipidosis) as a result of multiple alterations in lipid metabolism, among which are a lower β-oxidation and/or an increase in de novo synthesis [36]. Therefore, steatosis found in pathological studies is likely to be associated with the renal aging process and not with AD.

The increase in oxidative damage to proteins in the brain homogenate of male BALB/c_cenp_ mice of 12–13 months of age (Fig. 5) corresponds to the literature that describes it as a physiological event associated with the aging process [5] and is an element that supports the usefulness of these animals as a model of AD. In the pathophysiology of AD it’s known that oxidative stress plays an important role. The excess of free radicals and the special susceptibility of the nervous tissue to oxidative damage make it more vulnerable to attack by lipid membranes, proteins, RNA and nuclear DNA [39].

In the case of the finding of the decrease in brain size in mice aged 16–17 months (Fig. 6a), there isn’t evidence about the weight behaviour of this organ in aged mice, but it has been found post-mortem that the size of the brains of people with AD is lower than that of healthy people of the same age [40]. The decrease in brain weight is associated with the neuronal death described in AD [5], therefore this result contribute to collect experimental evidence to use aged BALB/c_cenp_ mice as model of dementia.

The degeneration found in the granular layer of the cerebellum of aged animals (Fig. 10) corresponds to the structural changes that have been described in the brain in the presence of dementia and specifically of AD in humans and nonhuman primates [41]. Although not many study references were found in mice, Jacobs et al. [42] demonstrated that transgenic mouse models carrying AD mutations (AbetaPP / PS1, APPswe / PS1dE9) have evidenced the role for the cerebellum in the pathophysiology of sporadic AD and its clinical manifestations. Early onset AD, especially from presenilin 1 mutations, exhibits cerebellar motor phenomena such as ataxia, as well as myoclonus or extrapyramidal symptoms. The fact that the cerebellum suffers the effects of this neurodegenerative pathology may be a side effect of the disease, or on the contrary, have a more specific importance in the degeneration process, or in its clinical consequences.

The cerebellum has an important function in the nervous system since it is responsible for the modulation of the cognitive and emotional part, in addition to its function in motor coordination. However, it is the first of these functions that has allowed in different studies the role of the cerebellum in neurodegenerative and neuropsychiatric disorders as in AD [41]. It is known that the cerebellum contributes to the cognitive and neuropsychiatric deficit in AD and in dementia in general. However, it is not clear in the literature whether a cerebellar lesion may be the cause or consequence of dementia [42].

The significant increase in the weight of the lungs (Fig. 6d) obtained in aged animals to 16–17 months may be related to the tumor lesions observed in lung histology in aged mice (Fig. 7). Lung disease in midlife may increase the risk of dementia. Many studies have showed that cognitive function is impaired in patients with lung disease with or without hypoxemia. Cerebral disturbance is noted in patients with lung disease and may be related to hypoxia in the brain [43].

Similar to our results, a significant increase in kidney weight (Fig. 6e) has previously been observed with aging in rodents that may be a result of a fibroblast accumulation as noted previously in renal tissue [44]. Lim et al. [44] reported that associated with aging in mice there is an increase in mesangial expansion and tubule interstitial fibrosis that is accompanied by inflammation, apoptosis and oxidative stress that together impair renal function. This deterioration in renal function was reflected through the glomerular degeneration found in the histology of this organ in BALB/c_cenp_ aged mice in our study (Fig. 9a). It has long been believed that kidney function is linked to brain activity. Clinical studies demonstrate that patients with a low glomerular filtration rate or structural or functional renal damage (chronic kidney disease) are more prone to cognitive impairment and AD, and the degree of cognitive impairment is closely related to kidney disease progression and renal failure. However, the mechanisms behind this linkage are unclear. For these reasons this is another element that supports the usefulness of BALB/c_cenp_ aged mice of 12–13 month of age as an AD model.

## Conclusions

The models of aged mice described for dementia present variability about the minimum age at which behavioral, pathological or biochemical symptoms of the disease take place [13, 14]. In spite of this variability it’s more advantageous to use aged mice with the minimum possible age because with very old animals the experiment becomes more expensive. In addition, the use of animals at the minimum age at which symptoms of dementia appear has the advantage that allows reducing the occurrence of fights among the groups that affect the health and welfare of animals. On the other hand at very advanced ages, animals are less motivates to participate in behavioral tests and other pathologies associated with the aging process appear that could affect the usefulness of the animal model. Therefore, we consider that it’s a very useful option to use animals 12–13 months of age where there are symptoms of cognitive deficiency, histopathological and biochemical elements characteristic of dementia and which were described earlier in this research.

## Data Availability

The data that support the findings of this study are available on request from the corresponding author on reasonable request.

## Abbreviations

AD: Alzheimer’s disease
ANOVA: One-way analysis of variance
EDTA: Ethylenediaminetetraacetic acid
FO: Familiar object
MDA: Malondialdehyde
NO: Novel object
PI: Preference index
SD: Standard deviation
TBARS: Thiobarbituric acid reactive substances
